# Complete genome sequence of *Pirellula staleyi* type strain (ATCC 27377^T^)

**DOI:** 10.4056/sigs.51657

**Published:** 2009-12-30

**Authors:** Alicia Clum, Brian J. Tindall, Johannes Sikorski, Natalia Ivanova, Konstantinos Mavrommatis, Susan Lucas, Tijana Glavina, Del Rio, Matt Nolan, Feng Chen, Hope Tice, Sam Pitluck, Jan-Fang Cheng, Olga Chertkov, Thomas Brettin, Cliff Han, John C. Detter, Cheryl Kuske, David Bruce, Lynne Goodwin, Galina Ovchinikova, Amrita Pati, Natalia Mikhailova, Amy Chen, Krishna Palaniappan, Miriam Land, Loren Hauser, Yun-Juan Chang, Cynthia D. Jeffries, Patrick Chain, Manfred Rohde, Markus Göker, Jim Bristow, Jonathan A. Eisen, Victor Markowitz, Philip Hugenholtz, Nikos C. Kyrpides, Hans-Peter Klenk, Alla Lapidus

**Affiliations:** 1 DOE Joint Genome Institute, Walnut Creek, California, USA; 2 DSMZ - German Collection of Microorganisms and Cell Cultures GmbH, Braunschweig, Germany; 3 Los Alamos National Laboratory, Bioscience Division, Los Alamos, New Mexico, USA; 4 Biological Data Management and Technology Center, Lawrence Berkeley National Laboratory, Berkeley, California, USA; 5 Oak Ridge National Laboratory, Oak Ridge, Tennessee, USA; 6 HZI – Helmholtz Centre for Infection Research, Braunschweig, Germany; 7 University of California Davis Genome Center, Davis, California, USA; * Corresponding author: Alla Lapidus

**Keywords:** prosthecate budding bacteria, developmental life cycle, Gram-negative, mesophile, *Planctomycetaceae*, *‘Planctomycetes’*, GEBA

## Abstract

*Pirellula staleyi* Schlesner and Hirsch 1987 is the type species of the genus *Pirellula* of the family *Planctomycetaceae*. Members of this pear- or teardrop-shaped bacterium show a clearly visible pointed attachment pole and can be distinguished from other *Planctomycetes* by a lack of true stalks. Strains closely related to the species have been isolated from fresh and brackish water, as well as from hypersaline lakes. Here we describe the features of this organism, together with the complete genome sequence and annotation. This is the first completed genome sequence of the order *Planctomyces* and only the second sequence from the phylum *Planctobacteria*/*Planctomycetes*. The 6,196,199 bp long genome with its 4773 protein-coding and 49 RNA genes is a part of the *** G****enomic* *** E****ncyclopedia of* *** B****acteria and* *** A****rchaea * project.

## Introduction

Strain ATCC 27377^T^ (= DSM 6068 = ATCC 27377) is the type strain of the species *Pirellula staleyi* and was originally isolated by James T. Staley in the early 1970s [1,2]. Due to superficially identified similarities with ‘*Pasteuria ramosa*’ in budding and rosette-forming, strain ATCC 27377^T^ was for several years considered to belong to the genus ‘*Pasteuria’* as the type strain of *P. ramosa* Metchnikoff 1888 [3]. However, Starr *et al*. [4] considered that this strain did not fit the original description of *P. ramosa* published by Metchnikoff in 1888 [3] and formally requested that the Judicial Commission rule that it should not be the type of *P. ramosa* Metchnikoff 1888. An Opinion was published by the Judicial Commission [5] fixing the type of *P. ramosa* Metchnikoff 1888 as the description of Metchnikoff as amended by Starr *et al*. [3]. At the same time Starr *et al* [3] also proposed that ATCC 27377^T^ be used as the type of a new species *Planctomyces staleyi.* In 1984 Schlesner and Hirsch re-assigned ATCC 27377^T^ to the new genus ‘*Pirella*’ [6] as the type strain to the only species ‘*Pirella staleyi*’ [6], but realized three years later that this genus name was as later homonym of *Pirella* Bainier 1883 [7], a fungus belonging to the *Mucorales*, and therefore illegitimate according to rule 51b of the *International Code of Nomenclature of Bacteria* [8,9]. In 1987 the strain received its currently validly published name *Pirellula staleyi*. *P. staleyi* and close relatives belong to the so called morphotype IV and are of interest because these organisms are usually attached to filamentous algae and cyanobacteria by a holdfast located at the distal end of the fascicle (the multifibrillar major appendage) or at the nonreproductive (nonbudding and nonpiliated) pole of the cell, if a fascicle is not present. *P. staleyi* is of further interest because of its life cycle (see below). It should be noted that members of the genus *Pirellula* (*P. staleyi*, *P. marina*) and other unnamed strains have been variously considered to be rapidly evolving (tachyletic) or ancient lineages. The transfer of *P. marina* to *Blastopirellula marina* and description of *Rhodopirellula baltica* [10] has called this interpretation into question, a theory that the growing number of genomes in the group may also be used to test.

Here we present a summary classification and a set of features for *P. staleyi* ATCC 27377^T^ (Table 1), together with the description of the complete genomic sequencing and annotation.

**Table 1:** Classification and general features of *P. staleyi* ATCC 27377^T^ in accordance with the MIGS recommendations [11]

**MIGS ID**	**Property**	**Term**	**Evidence code**
	Current classification	Domain *Bacteria*	
		Phylum *‘Planctomycetes’*	TAS [12]
		Class *Planctomycea*	TAS [12]
		Order *Planctomycetales*	TAS [13]
		Family *Planctomycetaceae*	TAS [13]
		Genus *Pirellula*	TAS [10]
		Species *Pirellula staleyi*	TAS [10]
		Type strain ICPB 4128	TAS [6]
	Gram stain	negative	TAS [6]
	Cell shape	pear or teardrop shaped	TAS [6]
	Motility	with flagella	TAS [6]
	Sporulation	sporulation has not been reported	NAS [6,14]
	Temperature range	mesophile, range has not been determined	TAS [14,15]
	Optimum temperature	20-25°C	TAS [14,16]
	Salinity	50% artificial seawater (100% ASW = 34.5 ‰ salinity)	TAS [6,14]
MIGS-22	Oxygen requirement	aerobic	NAS [6,14]
	Carbon source	Fucose, pectin, lactose, maltose**,** melibiose, raffinose, sucrose, and trehalose	TAS [6,14]
	Energy source	carbohydrates	TAS [6,14]
MIGS-6	Habitat	aquatic	TAS [17]
MIGS-15	Biotic relationship	free-living, but also attached to filamentous algae and cyanobacteria	TAS [17]
MIGS-14	Pathogenicity	non pathogenic	NAS
	Biosafety level	1	TAS [18]
	Isolation	fresh and brackish water	TAS [6,17]
MIGS-4	Geographic location	Lake Lansing, Michigan, USA	TAS [17]
MIGS-5	Sample collection time	early 1970s	TAS [17]
MIGS-4.1 MIGS-4.2	Latitude, Longitude	42.759, -84.399	NAS
MIGS-4.3	Depth	not reported	
MIGS-4.4	Altitude	not reported	

Evidence codes - IDA: Inferred from Direct Assay (first time in publication); TAS: Traceable Author Statement (i.e., a direct report exists in the literature); NAS: Non-traceable Author Statement (i.e., not directly observed for the living, isolated sample, but based on a generally accepted property for the species, or anecdotal evidence). These evidence codes are from the Gene Ontology project [19]. If the evidence code is IDA, then the property was directly observed for a living isolate by one of the authors, or an expert mentioned in the acknowledgements.

### Classification and features

To date, two strains of the species *P. staleyi* have been described in detail, ATCC 27377^T^ [6,9] and strain ATCC 35122 [15]. Strain ATCC 27377^T^ was isolated from the freshwater Lake Lansing, MI, USA either in 1973 or before [2]. Strain ATCC 35122 was isolated as a “white” subclone of strain ICPB 4232 from a similar habitat, the freshwater Campus Lake, Baton Rouge, LA, USA [15,20]. Both strains are identical in their 16S rRNA gene sequence [15]. Except for an agricultural soil bacterium clone (SC-I-28, AJ252628), and for the isolates ‘Schlesner 516’ and ‘Schlesner 670’ (X81940, X81948) [21], no 16S rRNA gene sequences above 85% sequence similarity were reported in Genbank. Environmental samples from metagenomic surveys also do not surpass 88-90% sequence similarity, indicating that members of the species are not heavily represented in the so far genomically screened habitats (as of August 2009). Interestingly, sequences most closely related to the planktonic, aerobic heterotroph *P. staleyi* have been reported from anoxic sediments of the productive freshwater lake Priest Pot, Cumbria, UK [22]. Also, *Pirellula*-like sequences have been recovered from DNA extracted from marine sediments in Pudget Sound [23] and marine snow [24].

**Figure 1:**
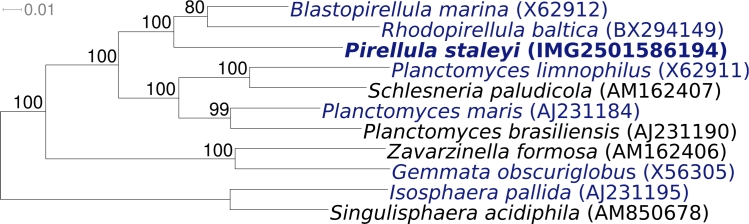
Phylogenetic tree highlighting the position of *P. staleyi* ATCC 27377^T^ relative to the other type strains within the family *Planctomycetaceae.* The tree was inferred from 1,316 aligned characters [25,26] of the 16S rRNA gene sequence under the maximum likelihood criterion [27] and rooted in accordance with the current taxonomy. The branches are scaled in terms of the expected number of substitutions per site. Numbers above branches are support values from 1,000 bootstrap replicates if larger than 60%. Lineages with type strain genome sequencing projects registered in GOLD [28] are shown in blue, published genomes in bold.

Figure 1 shows the phylogenetic neighborhood of *P. staleyi* ATCC 27377^T^ in a 16S rRNA based tree. The sequence of the sole 16S rRNA gene in the genome is identical to the previously published sequence generated from DSM 6068 (AJ231183).

**Figure 2:**
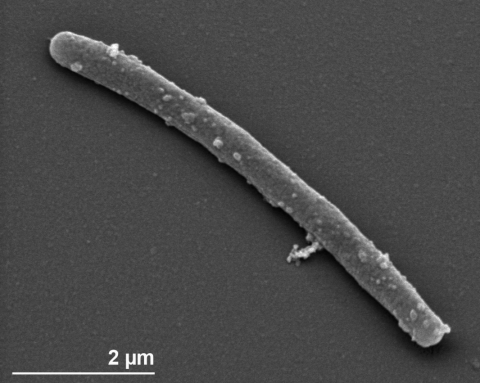
Scanning electron micrograph of *P. staleyi* ATCC 27377^T^

The cell size of strain ATCC 27377^T^ is 0.9-1.0 × 1.0-1.5 µm. The mature cell shape is teardrop- to pear-shaped, with the attachment pole slightly pointed (Figure 2). A fibrillar stalk shape and structure is absent. Crateriform structures are predominantly on the reproductive cell pole only. Occasionally, small crateriform structures may also be observed on the non-reproductive and nonpiliated pole of the cell opposite the budding site [17]. The position of the monotrichous flagellum is at the reproductive cell pole [6,10]. Strain ATCC 27377^T^ produces pigmented colonies and motile daughter and sessile mother cells [14].

A unique feature seen in both negatively stained cells and in thin-sectioned cells of strains ATCC 27377^T^ and ATCC 35122 is the occurrence of 'hump' protrusions including both cell wall and cytoplasm [15]. These protrude 50 to 111 nm from the cell and are 200to 260 nm in diameter measured at the base of the structure (from thin sections and negatively stained cells). One or two are visible per cell, and when two are visible these are distributed in a characteristic manner opposite to each other in the cell near the narrow pole [15]. They appear to conform to the definition of prosthecae as cellular appendages or extensions of the cell containing cytoplasm [15,29]. However, the prosthecae of strain ATCC 27377^T^ are distributed further from the narrow cell pole than in strain ATCC 35122 [15]. Functions proposed for the prosthecae include increasing surface area, reproduction, and stalk function [15].

The life cycle of *P. staleyi* has been described in great detail elsewhere [20]. Briefly, the mature bud develops a sheathed flagellum attached near the piliated pole (opposite the fascicle origin) and becomes a swarmer; the swarmer loses its flagellum and becomes a sessile mother cell (with a distal holdfast and eventually a fascicle at the pole opposite the piliated and budding pole); the mother cell develops a bud; etc [17,20].

Strain ATCC 27377^T^ hydrolyses casein, aesculin, gelatin and starch, but not DNA  [14]. It produces H_2_S from thiosulfate and is negative for lipase (pH 7) and phosphatidyl choline [14]. It utilizes fucose as carbon source, but not glycerol, glutamic acid, or chondroitin sulfate [14]. Contrary to the original description [2], the cells are gram-negative and do not utilize lyxose, D-ribose, fucose, L**-**rhamnose, fructose, or inulin as a carbon source. Additional characteristics include the following. Pectin, lactose, maltose**,** melibiose, raffinose, sucrose, and trehalose are utilized as carbon sources. The maximum salt tolerance is 50% artificial seawater (Lyman & Fleming, 1940), with 100% ASW corresponding to 3.5% salinity [4]. The cells are weakly inhibited by artificial light (2,400 lx). The following carbon sources are not utilized: adipate, citrate, I-alanine, I-glutamate, gluconate, and urea [4,7]. Strain ATCC 27377^T^ is resistant to ampicillin and penicillin (1000 µg ml^-1^), cephalotin (100 µg ml^-1^), streptomycin (500 µg ml^-1^) and cycloserine (100 µg ml^-1^), but not to tetracycline (10 µg ml^-1^ is lethal)  [14]. The primary sequence and secondary structure of the ribonuclease P RNA of strain *P. staleyi* ATCC 27377^T^ and other planctomycetes has been described in detail and has been evaluated for their suitability as a taxonomic marker [17].

### Chemotaxonomy

The cell envelope of strain *P. staleyi* ATCC 27377^T^ contains no peptidoglycan but consists almost entirely of protein. The cell wall amino acids (molar ratio) are threonine (3.0), glutamate (9.0), cysteine (3.6) and valine (1.7) [ 22]. Further details on the amino acids, NH_3_, hexosamine and neutral sugar contents of the cell envelope of strain ATCC 27377^T^ are published elsewhere [14]. The major fatty acids (relative %) are 16:0 (33.8), 18:1Δ9 (26.6), 20:1Δ11 (15.7), 17:1Δ9 (14.4), 17:0 (5.3), 16:1Δ9 (3.5), 18:0 (3.3), and 18:1Δ11 (2.0) [14]. The major polyamine is sym-homospermidine [50.2 µmol (g dry weight) ^–1^] [16]. The major respiratory lipoquinone present is MK-6. One of the major phospholipid present that has been identified is phosphatidylglycerol [14]. Other lipids have not been identified based on Rf values and staining behavior, indicating that novel lipids are an important constituent of the cell membrane. The production of spermidine distinguishes it from the closely related *R. baltica* DSM 10527^T^ and *B. marina* DSM 3645 ^T^.

## Genome sequencing and annotation

### Genome project history

This organism was selected for sequencing on the basis of its phylogenetic position, and is part of the *** G****enomic* *** E****ncyclopedia of* *** B****acteria and* *** A****rchaea * project. The genome project is deposited in the Genome OnLine Database [28] and the complete genome sequence is deposited in Genbank NOT YET. Sequencing, finishing and annotation were performed by the DOE Joint Genome Institute (JGI). A summary of the project information is shown in Table 2.

**Table 2:** Genome sequencing project information

**MIGS ID**	**Property**	**Term**
MIGS-31	Finishing quality	Finished
MIGS-28	Libraries used	One 8kb pMCL200 genomic library One 454 Pyrosequencing standard library and one Illumina library
MIGS-29	Sequencing platforms	ABI3730, 454 GS FLX, Illumina GA
MIGS-31.2	Sequencing coverage	10.6x Sanger; 20.4x Pyrosequencing
MIGS-30	Assemblers	Newbler version 1.1.03.24, PGA
MIGS-32	Gene calling method	Prodigal, GenePRIMP
	INSDC ID	N/A
	Genbank Date of Release	N/A
	GOLD ID	Gi02538
	NCBI project ID	29845
	Database: IMG-GEBA	2501533211
MIGS-13	Source material identifier	DSM 6068
	Project relevance	Tree of Life, GEBA

### Growth conditions and DNA isolation

*P. staleyi* ATCC 27377^T^, DSM 6068, was grown in DSMZ medium 595 (Caulobacter Medium, http://www.dsmz.de/microorganisms/media_list.php) at 26°C. DNA was isolated from 0.5-1 g of cell paste using MasterPure Gram-positive DNA Purification Kit (Epicentre MGP04100) with doubled volume (2 µl) lysozyme and incubated for one hour at 37°C according to Wu *et al*. [30].

### Genome sequencing and assembly

The genome was sequenced using a combination of Sanger, 454 and Illumina sequencing platforms. All general aspects of library construction and sequencing performed at the JGI can be found at the JGI website (http://www.jgi.doe.gov/). 454 Pyrosequencing reads were assembled using the Newbler assembler version 1.1.03.24 (Roche). Large Newbler contigs were broken into 6,869 overlapping fragments of 1,000 bp and entered into assembly as pseudo-reads. The sequences were assigned quality scores based on Newbler consensus q-scores with modifications to account for overlap redundancy and adjust inflated q-scores. A hybrid 454/Sanger assembly was made using the PGA (Paracel Genome Assembler) assembler. Possible mis-assemblies were corrected and gaps between contigs were closed by custom primer walks from sub-clones or PCR products. Illumina reads were used to improve the final consensus quality using an in-house developed tool (the Polisher). The error rate of the completed genome sequence is less than 1 in 100,000. The final assembly consists of 70,045 Sanger and 450,004 pyrosequence reads. Together all sequence types provided 31.0x coverage of the genome.

### Genome annotation

Genes were identified using Prodigal [31] as part of the Oak Ridge National Laboratory genome annotation pipeline, followed by a round of manual curation using the JGI GenePRIMP pipeline (http://geneprimp.jgi-psf.org/) [32]. The predicted CDSs were translated and used to search the National Center for Biotechnology Information (NCBI) nonredundant database, UniProt, TIGRFam, Pfam, PRIAM, KEGG, COG, and InterPro databases. Additional gene prediction analysis and functional annotation was performed within the Integrated Microbial Genomes - Expert Review (http://img.jgi.doe.gov/er) platform [33].

## Genome properties

**Figure 3:**
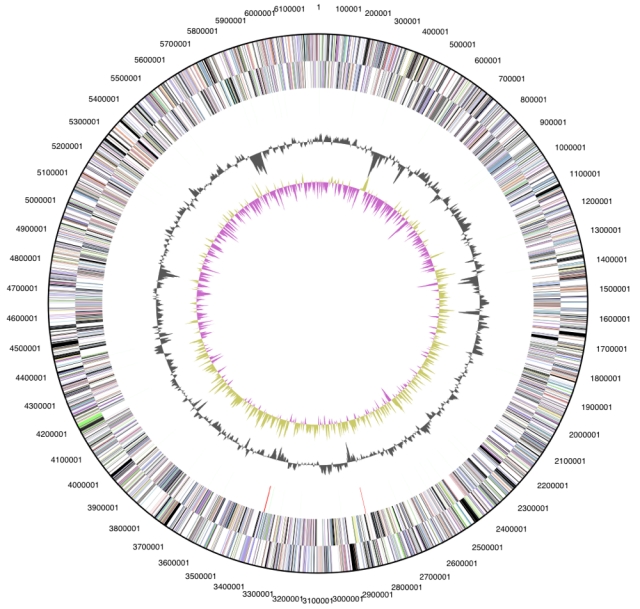
Graphical circular map of the genome. From outside to the center: Genes on forward strand (color by COG categories), Genes on reverse strand (color by COG categories), RNA genes (tRNAs green, rRNAs red, other RNAs black), GC content, GC skew.

The genome is 6,196,199 bp long and comprises one main circular chromosome with a 57.5% GC content (Table 3 and Figure 3). Of the 4,822 genes predicted, 4,773 were protein coding genes, and 49 RNAs. In addition, 56 pseudogenes were also identified. The majority of the protein-coding genes (54.5%) were assigned with a putative function while those remaining were annotated as hypothetical proteins. The distribution of genes into COGs functional categories is presented in Table 4.

**Table 3:** Genome Statistics

**Attribute**	Value	% of Total
Genome size (bp)	6,196,199	100.00%
DNA Coding region (bp)	5,362,662	86.55%
DNA G+C content (bp)	3,560,627	57.46%
Number of replicons	1	
Extrachromosomal elements	0	
Total genes	4,822	100.00%
RNA genes	49	1.02%
rRNA operons	1	
Protein-coding genes	4,773	98.98%
Pseudo genes	56	1.16%
Genes with function prediction	2,629	54.52%
Genes in paralog clusters	471	9.77%
Genes assigned to COGs	2,755	57.13%
Genes assigned Pfam domains	2,895	60.04%
Genes with signal peptides	1,414	29.32%
Genes with transmembrane helices	1,309	27.15%
CRISPR repeats	2	

**Table 4:** Number of genes associated with the general COG functional categories

Code	value	% of total	Description
J	149	3.1	Translation, ribosomal structure and biogenesis
A	2	0.0	RNA processing and modification
K	198	4.1	Transcription
L	153	3.2	Replication, recombination and repair
B	1	0.0	Chromatin structure and dynamics
D	21	0.4	Cell cycle control, mitosis and meiosis
Y	0	0.0	Nuclear structure
V	70	1.5	Defense mechanisms
T	197	4.1	Signal transduction mechanisms
M	164	3.4	Cell wall/membrane biogenesis
N	159	3.3	Cell motility
Z	1	0.0	Cytoskeleton
W	0	0.0	Extracellular structures
U	191	4.0	Intracellular trafficking and secretion
O	138	2.9	Posttranslational modification, protein turnover, chaperones
C	164	3.4	Energy production and conversion
G	169	3.5	Carbohydrate transport and metabolism
E	213	4.5	Amino acid transport and metabolism
F	64	1.3	Nucleotide transport and metabolism
H	135	2.8	Coenzyme transport and metabolism
I	96	2.0	Lipid transport and metabolism
P	150	3.1	Inorganic ion transport and metabolism
Q	60	1.3	Secondary metabolites biosynthesis, transport and catabolism
R	432	9.1	General function prediction only
S	285	6.0	Function unknown
-	2018	42.3	Not in COGs

## References

[1] Staley JT. The genus *Pasteuria*, p. 490-492. *In* M. Starr P,Stolp H, Trüper HG, A Balows A, HG Schlegel (eds), The prokaryotes. A handbook on habitats, isolation, and identification of bacteria. Springer-Verlag, Berlin, 1981.

[2] Staley JT Budding bacteria of the *Pasteuria-Blastobacter* group. Can J Microbiol 1973; 19:609-614 4122691 10.1139/m73-100

[3] Hirsch P Re-evaluation of *Pasteuria ramosa* Metchnikoff 1888, a bacterium pathogenic for *Daphnia* species. Int J Syst Bacteriol 1972; 22:112-116

[4] Starr MP Sayre RM Schmidt JM Assignment of ATCC 27377 to *Planctomyces staleyi* sp. nov. of *Pasteuria ramosa* Metchnikoff 1888 on and conservation the basis of type descriptive material. Request for an opinion. Int J Syst Bacteriol 1983; 33:666-671

[5] Judicial Commission of the International Committee on Systematic Bacteriology Opinion 61 Rejection of the type strain of *Pasteuria ramosa* (ATCC 27377) and conservation of the species *Pasteuria ramosa* Metchnikoff 1888 on the basis of the type descriptive material. Int J Syst Bacteriol 1986; 36:119

[6] Schlesner H Hirsch P Assignment of ATCC 27377 to *Pirella* gen. nov. as *Pirella staleyi* comb. nov. Int J Syst Bacteriol 1984; 34:492-495

[7] Bainier G Observations sur les mucorinées. Ann Sci Nat Bot Sér VI 1883; 15:70-104

[8] Lapage SP, Sneath PHA, Lessel EF, Skerman VBD, Seeliger HPR, Clark WA. International code of nomenclature of bacteria (1975 revision). American Society for Microbiology, Washington, DC.

[9] Schlesner H Hirsch P Rejection of the genus name *Pirella* for pear-shaped budding bacteria and proposal to create the genus *Pirellula* gen. nov. Int J Syst Bacteriol 1987; 37:441

[10] Schlesner H Rensmann C Tindall BJ Gade D Rabus R Pfeiffer S Hirsch P Taxonomic heterogeneity within the lanctomycetales as derived byDNA–DNA hybridization, description of *Rhodopirellula baltica* gen.nov.,sp.nov.,transfer of *Pirellula marina* to the genus *Blastopirellula* gen.nov.as *Blastopirellula marina* comb.nov. and emended description of the genus *Pirellula.* Int J Syst Evol Microbiol 2004; 54:1567-1580 10.1099/ijs.0.63113-0 15388712

[11] Field D Garrity G Gray T Morrison N Selengut J Sterk P Tatusova T Thomson N Allen MJ Angiuoli SV The minimum information about a genome sequence (MIGS) specification. Nat Biotechnol 2008; 26:541-547 10.1038/nbt1360 18464787 PMC2409278

[12] Cavalier-Smith T The neomuran origin of archaebacteria, the negibacterial root of the universal tree and bacterial megaclassification. Int J Syst Evol Microbiol 2002; 52:7-76 11837318 10.1099/00207713-52-1-7

[13] Schlesner H Stackebrandt E Assignment of the genera *Planctomyces* and *Pirella* to a new family *Planctomycetaceae* fam. nov. and description of the order *Planctomycetales* ord. nov. Syst Appl Microbiol 1986; 8:174-176

[14] Schlesner H Rensmann C Tindall BJ Gade D Rabus R Preiffer S Hirsch P Taxonomic heterogeneity within the *Planctomycetales* as derived by DNA-DNA hybridization, description of *Rhodopirellula baltica* gen. nov., sp. nov., transfer of *Pirellula marina* to the genus *Blastopirellula* gen. nov. as *Blastopirellula marina* comb. nov. and emended description of the genus *Pirellula.* Int J Syst Evol Microbiol 2004; 54:1567-1580 10.1099/ijs.0.63113-0 15388712

[15] Butler MK Wang J Webb RI Fuerst JA Molecular and ultrastructural confirmation of classification of ATCC 35122 as a strain of *Pirellula staleyi.* Int J Syst Evol Microbiol 2002; 52:1663-1667 10.1099/ijs.0.02167-0 12361271

[16] Griepenburg U Ward-Rainey N Mohamed S Schlesner H Marxsen H Stackebrandt E Auling G Phylogenetic diversity, polyamine pattern and DNA base composition of members of the order *Planctomycetales.* Int J Syst Bacteriol 1999; 49:689-696 10319492 10.1099/00207713-49-2-689

[17] Starr MP Sayre RM Schmidt JM Assignment of ATCC 27377 to *Planctomyces staleyi* sp. nov. and Conservation of *Pasteuria ramosa* Metchnikoff 1888 on the Basis of Type Descriptive Material: Request for an Opinion. Int J Syst Bacteriol 1983; 33:666-667

[18] Anonymous. Biological Agents: Technical rules for biological agents www.baua.de TRBA 466.

[19] Ashburner M Ball CA Blake JA Botstein D Butler H Cherry JM Davis AP Dolinski K Dwight SS Eppig JT Gene ontology: tool for the unification of biology. The Gene Ontology Consortium. Nat Genet 2000; 25:25-29 10.1038/75556 10802651 PMC3037419

[20] Tekniepe BL Schmidt JM Starr P Life cycle of a budding and appendaged bacterium belonging to morphotype IV of the *Blastocaulis-Planctomyces* group. Curr Microbiol 1981; 5:1-6 10.1007/BF01566588

[21] Ward N Rainey FA Stackebrandt E Schlesner H Unravelling the extend of diversity within the order *Planctomycetales.* Appl Environ Microbiol 1995; 61:2270-2275 7793948 10.1128/aem.61.6.2270-2275.1995 PMC167499

[22] Miskin IP Farrimond P Head IM Identification of novel bacterial lineages as active members of microbial populations in a freshwater sediment using a rapid RNA extraction procedure and RT-PCR. Microbiology 1999; 145:1977-1987 10.1099/13500872-145-8-1977 10463164

[23] Gray JP Herwig RP Pjylogenetic analysis of the bacterial communities in marine sediments. Appl Environ Microbiol 1996; 62:4049-4059 8899989 10.1128/aem.62.11.4049-4059.1996 PMC168226

[24] DeLong EF Franks DG Yayanos AA Phylogenetic diversity of aggregate-attached vs. free-living marine bacterial assemblages. Limnol Oceanogr 1993; 38:924-934

[25] Castresana J Selection of conserved blocks from multiple alignments for their use in phylogenetic analysis. Mol Biol Evol 2000; 17:540-552 10742046 10.1093/oxfordjournals.molbev.a026334

[26] Lee C Grasso C Sharlow MF Multiple sequence alignment using partial order graphs. Bioinformatics 2002; 18:452-464 10.1093/bioinformatics/18.3.452 11934745

[27] Stamatakis A Hoover P Rougemont J A Rapid Bootstrap Algorithm for the RAxML Web Servers. Syst Biol 2008; 57:758-771 10.1080/10635150802429642 18853362

[28] Liolios K Mavromatis K Tavernarakis N Kyrpides NC The Genomes On Line Database (GOLD) in 2007: status of genomic and metagenomic projects and their associated metadata. Nucleic Acids Res 2008; 36:D475-D479 10.1093/nar/gkm884 17981842 PMC2238992

[29] Staley JT *Prosthecomicrobium* and *Ancalomicrobium*: New prosthecate freshwater bacteria. J Bacteriol 1968; 95:1921-1942 4870285 10.1128/jb.95.5.1921-1942.1968 PMC252228

[30] Wu M, Hugenholtz P, Mavromatis K, Pukall R, Dalin E, Ivanova N, Kunin V, Goodwin L, Wu M, Tindall BJ, *et al.* A phylogeny-driven genomic encyclopedia of Bacteria and Archaea. *Nature, in revision*10.1038/nature08656 PMC3073058 20033048

[31] Anonymous. Prodigal Prokaryotic Dynamic Programming Genefinding Algorithm. Oak Ridge National Laboratory and University of Tennessee 2009 http://compbio.ornl.gov/prodigal

[32] Pati A, Ivanova N, Mikhailova N, Ovchinikova G, Hooper SD, Lykidis A, Kyrpides NC. GenePRIMP: A Gene Prediction Improvement Pipeline for microbial genomes. (Submitted) 2009 10.1038/nmeth.1457 20436475

[33] Markowitz VM Mavromatis K Ivanova NN Chen IMA Chu K Kyrpides NC Expert IMG ER: A system for microbial genome annotation expert review and curation. Bioinformatics 2009; 25:2271-2278 10.1093/bioinformatics/btp393 19561336

